# Supplementary material for: Are carbon emissions trading and green financial instruments synergistic? -Comprehensive quantitative research based on content analysis
Source: PLoS One. 2024 Mar 7;19(3):e0298601. doi: 10.1371/journal.pone.0298601 (PMC10919601; doi:10.1371/journal.pone.0298601)
Supplement: S1 Data — (ZIP) [file pone.0298601.s001.zip › Data/地级市Z_F工作报告.zip!/║■─╧/╙└╓▌╩╨/└┤╫╘╣1⁄2╓┌║┼í╛┬φ┐╦╩2╛▌═°í┐_2015.docx]

各位代表：

　　现在，我代表市人民政府向大会作政府工作报告，请予审议，并请各位政协委员和列席人员提出意见。

　　一、奋力攻坚克难，2014年工作取得新成效

　　过去一年，在省委、省政府和市委的坚强领导下，我们认真贯彻落实 “四化两型”总战略、“三量齐升”总要求和“四个三”的决策部署，牢牢把握“稳中求进、改革创新”总基调，努力克服经济下行等诸多困难，积极应对经济发展新常态，全市经济实现逆势进位，社会大局保持和谐稳定。

　　克服下行压力，发展势头稳定向好。经济保持较快增长。完成地区生产总值1299.93亿元、增长9.9%，固定资产投资1303.54亿元、增长21.3%，社会消费品零售总额423.27亿元、增长12.9%，财政总收入113.39亿元、增长13%，城乡居民收入达到20175元、9873元，分别增长8.9%和11.4%。主要经济指标增速高于全省平均水平，在全省排位较大幅度前移。结构调整取得实效。产业结构呈现新变化，三次产业比调整为21.9∶37.8∶40.3，一产业下降0.5个百分点，三产业提高0.8个百分点。传统产业整合提升步伐加快，电子信息、新能源、新材料、生物医药等新兴产业发展提速。瑞翔新材料、千山医疗器械、凯盈科技、广隆科技等高新技术企业建成投产。发展后劲持续增强。“十百千工程”建设扎实推进，全年投资过亿元项目203个，增加73个，其中新开工投资过亿元项目126个，增加51个。网络购物、信息、汽车、旅游等消费持续旺盛。招商引资成效显著，实际利用外资7.51亿美元、增长20.1%，到位内资178.1亿元、增长12.4%，江华成为全市县域承接产业转移排头兵。对外贸易大幅增长，进出口总额完成9.39亿美元、增长108.9%，增幅居全省第一。年末金融机构存款余额1338.19亿元、增长14.1%，贷款余额641.37亿元、增长15.7%。

　　实施“三大突破”，瓶颈制约逐步缓解。工业发展提质加速。完成规模以上工业增加值297.97亿元、增长12.2%，增幅连续7个月排全省第一，被列为全省加速推进新型工业化十大亮点之一。新增规模工业企业106家、年产值过亿元企业32家。零陵烟厂完成产值60.5亿元，对全市工业经济发展贡献突出。产业园区新入园企业134家，新建标准厂房201万平方米。城镇面貌大为改观。确立中心城区“一主三副”城市构架和“一江两岸、东扩西跨、南北联城、中部崛起”扩容思路，实施三年扩容提质计划，湘江风光带、城南大道、湘江东路、湘江西路、阳明大道、芝山北路等道路建设取得实效，20条断头路有15条基本拉通，98条背街小巷和29个农贸市场改造、55个城中村整治基本完成，控违拆违取得阶段性成效，闲置土地清理稳步推进。城市创建工作实现重大突破，创建国家卫生城市顺利通过全国爱卫办综合评审，带动和促进了国家历史文化名城申报和国家交通管理模范城市、国家森林城市的创建。文明卫生县城和品质活力乡镇建设取得成效。永州上榜中国幸福城市20强。基础条件明显改善。交通建设迈出新步伐，湘桂高铁车次加密，二广高速永州至广东段、祁冷一级公路通车，207国道双牌大山隧道贯通，干线公路新改建260公里。零陵机场迁建、洛湛铁路益永段扩能改造等项目前期工作取得进展。能源建设取得实效，投资80亿元的神华国华永州火电一期工程开工建设，成为我市迄今投资额最大的能源项目，风电项目建设和新一轮农网改造升级加快推进。农田水利建设得到加强，涔天河水库扩建工程和库区移民工作扎实推进，芦江水库、金钩挂水库开工建设，63座小二型病险水库除险加固、10个中小河流治理和7个小农水重点县项目完成年度任务，完成高标准农田建设7万亩、中低产田改造3.84万亩。双牌获批国家农业综合开发县。

　　深化“三项改革”，发展活力持续释放。行政管理体制改革全省领先。建立市级权力清单、负面清单、监管清单“三单管理”制度。商事制度“先照后证”、“三证合一”改革全面启动，在全省率先发出第一张“三证合一”证照。公共资源交易体制改革顺利实施。农村产权制度改革扎实推进。土地确权工作在全省率先全面推开并基本完成。市、县、乡三级土地流转服务平台初步建立，全年新增土地流转面积116.7万亩、增长28%。农村新型经营主体发展迅速，培育农民专业合作社1736家、专业大户和家庭农场1.5万个。全省农村土地承包经营权抵押融资试点在新田推开。社会治理机制改革落地生根。“智慧永州”综合运用及电子政务公开平台建设项目正式启动，中心城区街道服务职能和工作人员下移，社区合并、职能调整和网格化布局基本完成，县城网格化建设全面铺开，农村网格化试点开始启动。市属国有企业改革任务基本完成。宁远财政绩效管理工作荣获全国先进。江永和祁阳农村信用社改制为农村商业银行。省定改革年度目标任务全面完成。

　　坚持“三项优先”，幸福指数不断提升。生态建设力度加大。编制完成全市主体功能区规划，江华、蓝山、新田、宁远、双牌纳入国家第一批主体功能区建设试点。扎实推进“绿色永州”建设，完成植树造林53万亩，封山育林70万亩。蓝山、江华、宁远湘江源头区域列为国家第一批生态文明先行示范区，日月湖、紫水获批国家湿地公园。湘江保护和湘江污染防治“三年行动计划”全面实施，大气污染治理扎实推进，农村环境综合整治取得实效。省定节能减排任务全面完成。教育事业全面发展。加大资金投入力度，新增城区义务教育学位1.58万个，其中中心城区5700个，建成义务教育合格学校83所、农村公办幼儿园28所，新增教师周转房1000套。推行校长负责制，名校名师名校长建设取得进展，我市高考一本上线人数增幅居全省第一。高等教育、职业技术教育稳步发展，祁阳获批全国农村职业教育和成人教育示范县。社会就业更加充分。全年新增城镇就业6.5万人、失业人员再就业2.5万人、农村劳动力转移就业7.1万人，城镇零就业家庭动态就业援助率达到100%。积极推进省级创业型城市建设，创业孵化基地和创业示范基地建设取得新的成效。

　　各项事业全面进步。民生改善成效显著，用于民生的支出占财政总支出的77.9%。社会保障得到加强，五项社会保险参保人数639.7万人次，发放社会保障卡271万张。城市低保对象月人均253.08元，农村低保对象月人均115.9元，五保对象年分散供养标准为2769.5元，均超过省考核标准。新农合参合率达到99.13%。医药卫生改革深入推进，基本药物制度稳步实施，县级公立医院改革试点初见成效。科技创新取得新进步，申请专利2010件，授权专利1020件，新增高新技术企业5家。文化惠民工程深入实施，农村公共文化服务加强，广播电视“村村通”工程完成1.6万套、覆盖5万户，农村电影放映6万场次。人口计生工作保持全省先进，祁阳、江永获得“全国计划生育优质服务先进单位”。群众体育和竞技体育协调发展。工会、共青团、妇联、工商联、贸促会、侨联等群团组织作用充分发挥。扶贫开发、移民工作成效显著。审计、统计、人防、民族宗教、外事侨务、新闻出版、史志、气象、地震、档案、对台、老龄、关工委、残联等工作都取得新的进步。

　　推进“三项统筹”，协调发展能力增强。小康建设步伐加快。突出分类指导，发挥优势特色，全面小康总体实现程度提高3个百分点以上。推进精准扶贫、科学扶贫、内源扶贫，全年减少贫困人口13.1万人，三类县贫困发生率降低4%以上。农村经济稳步发展，粮食、蔬菜和水果生产获得丰收，烤烟收购61.4万担，祁阳获全国粮食生产先进县，道县蔬菜园获批国家级蔬菜标准园，新田荣获中国天然富硒农产品之乡称号。农业产业化步伐加快，新增省级龙头企业10家、省著名商标11个，熙可食品获省长质量奖，江永香姜获国家地理标志证明商标。新农村建设深入推进，13个示范村、13个城乡统筹试点镇建设取得新成效，创建美丽乡镇10个、美丽乡村20个，启动建设秀美村庄5329个。文化旅游融合发展。“古城名山”建设扎实推进，阳明山被确定为海峡两岸交流基地，九嶷山被评为国家级风景名胜区。濂溪故里、蘋州书院成功创建3A景区，涧岩头村、上甘棠村、龙溪村、坦田村被授予全国传统村落文化保护利用示范村。全年接待国内外游客3040.6万人次、增长24.1%，实现旅游收入157.1亿元、增长24.5%。惠民实事全面落实。省定为民办实事任务全面完成。市里确定的六件惠民实事全面落实，实施棚户区改造项目35个，开工15782户，中心城区小区集中整治工作基本完成，138个保障房项目进展顺利，安置小区建成3个、开工建设17个。农村生活垃圾无害化处理率逐步提高，“禁养区、限养区、适养区”划定基本完成。建成放心食用油示范店73家、放心米粉店36家。完成“五小”水利工程8560处，新解决30.1万农村人口饮水安全问题。安全生产考核排名全省第二。政法队伍建设满意度评价全省第一，综治民调综合排名全省第三。“六五”普法全面开展。

　　强化落实执行，依法行政得到加强。自觉接受市人大及其常委会的法律监督、工作监督和市政协的民主监督，充分吸纳民主党派、工商联、无党派人士的意见建议，全年共办理代表大会议案2件、人大代表建议176件，政协委员提案224件，办复率100%。创建国家卫生城市议案办理圆满成功，闲置土地清理处置议案办理效果明显，启动207国道零陵至双牌段改造工程、加快推进农村土地流转等8件代表建议办理较好。市委、市政府主要领导及市政协领导领衔督办的15件重点提案办理全面落实。市本级行政审批事项削减率达27.4%，建设工程项目并联审批制度深入推进。大力开展“项目建设年”和“企业服务年”活动，一批群众和企业反映强烈的突出问题得到解决。扎实开展党的群众路线教育实践活动，大力整治“四风”，政府公信力和干部执行力得到较大提升。

　　国防动员和后备力量建设成效明显。人民武装和国防动员工作制度进一步健全，双拥共建和“双带双促”活动深入推进，后备力量建设规模、结构布局进一步优化。驻永解放军、武警部队和民兵预备役人员为全市经济社会发展作出了积极贡献。

　　各位代表，过去一年所取得的成绩，是省委、省政府正确领导、高度重视的结果，是市委精心谋划、科学决策的结果，是市人大、市政协加强监督、大力支持的结果，是全市上下奋力攻坚、团结拼搏的结果。在此，我谨代表市人民政府，向全市人民，向驻永部队、武警官兵，向中央、省驻永单位，向所有关心支持永州发展的各级领导、各界朋友表示衷心的感谢！

　　在肯定成绩的同时，我们也清醒地认识到工作中存在的困难和问题，主要是：经济下行压力持续加大，地区生产总值、固定资产投资、社会消费品零售总额等指标增幅与年初目标比还有差距; 大项目、好项目偏少，园区建设水平不高，部分行业和企业生产经营困难；资金投入、项目用地、劳动用工等瓶颈制约依然严重，经济运行中还存在一些风险和隐患；部分群众生活比较困难，全面小康建设任务繁重; 少数政府工作人员作风不实，推动力和执行力有待加强。对此，我们一定高度重视，采取有效措施，认真加以解决。

　　二、清醒研判形势，主动适应经济发展新常态

　　2015年是全面深化改革的攻坚之年，是全面推进依法治国的开局之年，也是“十二五”收官和“十三五”谋划之年。做好今年的各项工作，必须立足永州实际，清醒研判形势，准确把握中央的重大战略判断，全面认识新常态，积极应对新常态，努力实现新常态下的新发展。

　　必须清醒看到，永州正处于迎难而进的攻坚期。纵观国际局势，世界经济仍在深度调整，总体复苏疲弱态势难有明显改观。放眼国内形势，我国已进入以增长速度换挡、发展方式转变、经济结构调整、发展动力转换为主要特征的新常态，困难和挑战层出不穷。环顾省内周边，长株潭城市群、环洞庭湖区域已站在更高的发展起点，大湘西地区扶贫开发热潮全面兴起，同属湘南板块的衡阳、郴州，同处“千亿俱乐部”的兄弟市州发展势头强劲，给我们带来很大的竞争压力。审视自身发展，我市经济总量不大、结构不优，质量不高，特别是在新常态下，经济发展面临的不确定、不稳定因素增多，部分行业和企业生产经营困难，房地产市场持续低迷，“土地财政”难以为继，政府性债务及金融等领域存在风险和隐患，实现稳增长形势严峻、任务艰巨。

　　必须清醒看到，永州正处于抢抓机遇的关键期。新常态带来新挑战，也蕴含新机遇。新常态下，经济增速虽然放缓，但增长更趋平稳，动力更为多元，预期更加稳定，有利于加快产业转型升级，提高经济发展质量。新常态下，简政放权力度进一步加大，市场决定性作用进一步增强，有利于释放市场主体的活力。新常态下，国家更加注重区间调控、定向调控，重视中西部地区发展，关注铁路、信息网络、能源建设、农田水利、生态治理、健康养老等重点领域和薄弱环节，有利于我们争取国家项目和资金支持。新常态下，国家更加重视优化区域发展布局，重点实施“一带一路”、京津冀协同发展、长江经济带三大战略，特别是在中央对湖南 “一带一部”的科学定位中，我市正处于“一带一部”重要节点和对接东盟黄金通道上，有利于我们加快承接对接，扩大开放引进。

　　必须清醒看到，永州正处于稳定向好的发展期。从国内形势看，我国发展仍处于大有作为的重要战略机遇期，经济发展总体向好的基本面没有改变，这为我市发展提供了良好的外部环境。经过多年打基础增后劲，支撑全市经济发展的利好因素不断集结，涔天河水库扩建、神华国华永州火电等一批重大项目上马推进，零陵烟厂、猎豹汽车等骨干企业形势向好，大批招商引资企业相继发力，县域经济活力明显增强，多点支撑、多极发展态势正在形成。各级各部门凝心聚力、和衷共济，全市上下风清气正、心顺劲足，改革创新的动能越来越足，加快发展的氛围越来越浓。

　　新的一年，机遇与挑战同在，希望与困难并存，机遇难得，希望可期。我们要认清发展形势，保持清醒头脑，既要增强忧患意识，更要坚定发展信心，既要突出问题导向，更要善于打开局面，既要坚持底线思维，更要强化创先争优，进一步开创全市经济社会发展新局面。

　　2015年工作总体要求是：以党的十八大、十八届三中、四中全会和中央、省委、市委经济工作会议精神为指引，坚持稳中求进的工作总基调，以提高发展质量和效益为中心，以全面建成小康社会为总揽，主动适应新常态，深入实施“四个三”工作部署，着力推进改革攻坚，着力强化创新驱动，着力破解发展难题，着力保障改善民生，实现经济平稳健康发展和社会和谐稳定，不断提升软实力，加快建设品质活力永州。

　　主要预期目标是：地区生产总值增长9%，规模工业增加值增长12%，固定资产投资增长20%，社会消费品零售总额增长13%，财政总收入增长12%，城乡居民收入分别增长10%和12%，城镇登记失业率控制在4%以内，人口自然增长率控制在9‰以内，全面小康实现程度提升3个百分点，省里下达的节能减排等约束性指标全面达标。

　　实现上述预期目标，必须牢牢把握以下六个方面：

　　坚持把加快发展作为第一要务。立足发展不快、发展不足的最大市情，顺应人民过上更好生活的殷切期盼，始终突出发展第一要务，积极有为、主动作为，力求稳中求进，进中求快，快中求好，又好又快，全面完成“十二五”规划目标任务。

　　坚持把项目建设作为第一抓手。实施重大项目带动战略和“1511”投资计划，着力抓好一批补短板、促升级、惠民生的重大项目，以大项目、好项目带动大投入，推动大发展，努力营造齐心协力上项目、全力以赴促发展的浓厚氛围。

　　坚持把改革开放作为第一动力。以深化改革破解发展难题，深入推进“3+2”改革，走出全面深化改革的“永州路径”，加速释放改革红利。以扩大开放赢得发展先机，加快推进示范区建设，发展开放型经济，努力构建全方位、多层次、宽领域的大开放格局。

　　坚持把改善民生作为第一追求。以提升群众幸福指数为出发点和落脚点，优先保障民生投入，优先安排民生工程，优先解决民生问题，让改革发展的成果更多更公平地惠及全市人民。

　　坚持把创优环境作为第一责任。适应新常态，必须从过去靠各类优惠政策形成竞争优势，切实转换到通过优化发展环境来提升软实力和竞争力，加快形成统一透明、有序规范、可以预期的良好市场环境。

　　坚持把法治建设作为第一保障。以建设法治永州为目标，切实把法治精神、法治意识渗透到依法治市的全过程，为加快建设品质活力永州提供坚实的法治保障。

　　三、坚持稳中求进，推动科学发展迈上新台阶

　　做好今年政府工作，必须继续坚定不移按照“四个三”的工作部署，全面实施八项计划，着力在深化深入、提高提升、落地落实上下功夫，着力在重点工作、发展难题、关键举措上求突破，进一步提升全市经济发展的整体水平。

　　（一）着力培育新增长点，促进经济稳定健康发展

　　大力推进新型工业化。高举新型工业化第一推动力旗帜，研究完善产业布局，构建现代产业体系，着力实施规模工业企业转型升级计划、全民创新创业计划，实现规模工业总产值突破1000亿元，新入统规模企业100家，新增主营业务收入5亿元以上企业10家。推进产业转型升级。加快改造提升传统产业，打造粮油加工、果蔬加工、烟草、建材四大百亿产业，发展千亿矿业经济。大力培育战略性新兴产业，重点发展先进装备制造、电子信息、生物医药、新能源、新材料等产业，年内先进装备制造和电子信息产业实现过百亿目标。着力培育龙头企业。全力支持零陵烟厂技改扩能，力争完成产值80亿元以上。帮助猎豹汽车CS10尽快下线量产，抢占市场。抓好神华国华永州火电、台湾高科园、烟草产业园、江华稀土、祁阳海螺水泥二期等重大项目建设，力争菲斯克新能源汽车、象外科技、立德绿色建筑、台湾光塑、崧顺电子等项目尽早开工建设。加快产业园区建设。大力实施百亿工业园区推进计划，全年规模工业总产值过百亿园区达到2个。加快永州国家农业科技园建设，力争3至5年内建成百亿园区。全力推进创新创业园区建设“135”工程，11个县区和永州经开区要引进企业300家，新建标准厂房200万平方米以上。改革园区管理体制，减少园区管理层级和环节，赋予园区县级政府管理权限，强化对园区工作的评价考核。扶持小微企业成长。深入实施“扶助小微企业专项行动”，落实各项政策措施，发挥创业基地作用，支持青年创新创业园发展，做实做强中小企业担保平台，全年新增小微企业1300家以上。

　　大力发展现代农业。做强优势产业。稳定大宗农产品生产，确保粮食面积825万亩，水果150万亩，蔬菜230万亩，烤烟25万亩。深化与温氏集团等龙头企业的合作，推动生猪、肉牛、奶牛等养殖基地建设。大力发展粮油、果蔬、油茶、茶叶、毛竹、家具、畜禽等重点产业，新增农产品加工龙头企业20家以上、年销售收入过亿元企业5家以上。做优特色品牌。积极创建省级农业标准化示范县，建设市、县级农业标准化示范基地88个，新发展“三品一标”农产品70个，新增“三品一标”认证单位5个以上。扎实推进舜皇山土猪、阳明红牛、九嶷山兔、东安鸡等地方特色养殖，打好永州异蛇、新田富硒、江永五香、蓝山三峰茶、道县脐橙等地方特色品牌，新增省级以上著名商标10个以上。做实优质服务。统筹整合涉农资金，创新农业投融资机制。完善农业服务体系，加强农机、供销、植保、农资、农技等社会化服务组织建设。提升农业综合生产能力，抓好农业综合开发、国土整理、现代烟草农业示范等项目，完成高标准农田建设11.3万亩。

　　大力提升现代服务业。发展商贸流通产业。着力抓好冷水滩河东、河西城市综合体、华天城、万商红等项目。继续抓好农贸市场标准化改造。引进发展第三方物流和电商物流平台，培育大型物流和电商龙头企业。积极发展新型服务业态。大力培育电子商务、信息咨询、文化创意、健康养老等新型服务业，借助互联网、物联网，促进传统服务业转型升级。全面落实中央和省里促进消费的政策措施，推动信息、旅游、汽车等消费热点持续升温。针对房地产业持续下滑态势，制定出台扶持政策，引进龙头开发企业，促进房地产业平稳健康发展，稳定增加住房消费。加快发展文化旅游产业。以打造“古城名山”为龙头，加强旅游资源和精品线路策划开发，加快零陵古城、江华神州瑶族文化博览园等项目建设。积极推进等级旅游景区创建工作，力争九嶷山创5A，江永勾蓝瑶寨、双牌桐子坳创3A，宁远三分石创省级生态旅游示范区，启动阳明山国家旅游度假区创建工作。利用湘桂高铁和二广高速开通的机遇，加大与高铁沿线和珠三角地区的区域旅游合作。

　　（二）深化改革开放，不断增强经济发展动力

　　突出改革推动。深化行政管理体制改革。进一步简政放权，转变政府职能，有序推进市县政府机构改革。完善权力清单、负面清单、监管清单和责任清单“四单管理”。优化政务服务中心和政府服务网络两个窗口，推动审批流程再造。深化“先照后证”、“三证合一”商事制度改革，加强改革后的市场监管。深化农村产权制度改革。上半年完成农村土地承包经营权确权颁证工作，年底前基本完成县区、乡镇、村三级农地利用规划编制。规范农村土地流转平台，完善服务体系。全面推行农村土地承包经营权抵押贷款。深化社会治理机制改革。加快“智慧永州”建设，推进网格化管理与信息化支撑的深度融合，全面完成市县城区网格化建设任务，推进农村网格化试点。加快社区标准化建设，增强社区服务和管理能力。深化国资国企改革。全面完成国有企业改制扫尾工作，妥善处理企业办社会职能分离移交、改制企业后续管理等遗留问题。研究完善新形势下国资监管体制。推进财税和投融资体制改革。加强财源培植，坚持依法治税，规范完善财税运行机制，强化财政性资金绩效管理，清理规范税收优惠政策。对政府性债务类别和规模实行“双控”管理，完善政府投融资平台管理和融资机制，推进平台公司转型发展。探索PPP、资产证券化、股权融资等投融资模式，选择一批项目开展PPP试点，鼓励社会资本参与公共服务领域的建设和运营。推进金融改革创新。建立完善地方金融体系，争取新引进１家以上股份制银行，推行“一县两行”模式，新引进2家以上村镇银行，完成新的潇湘农商银行组建挂牌，推进县级农村信用社改制成立农商银行。引进保险资金入永投资项目。规范发展小额贷款公司、融资性担保公司。扩大直接融资，争取1家企业首发上市、１家企业新三板挂牌。加强信用体系建设，优化金融生态环境，争创１个省级金融安全区、1至2个市级金融安全区。加强金融监管，制定应对预案，有效防范金融和债务风险。此外，还要切实抓好教育、文化、医药卫生体制改革、要素市场改革、社会信用体系建设和扩权强镇、并乡合村、小城镇管理体制改革等其他领域改革。

　　突出开放带动。着力开展大招商。夯实招商基础，精心建好项目库和客商库，围绕全市资源优势和发展重点，策划包装一批大项目、好项目。创新招商方式，继续开展小分队精准招商，每个市直招商小分队年内至少引进1个投资5000万元以上的工业或商贸物流项目；开展商会招商，发挥异地商会作用，打响“永商”品牌；推进物流招商，以物流业的发展带动上下游产业的配套，吸引厂商、电商投资落户。完善招商项目联席会议机制，加强项目跟踪服务，提高项目履约率、开工率和资金到位率。积极推进大合作。以湘南国家级承接产业转移示范区为平台，深化与珠三角、港澳台、东盟、北部湾以及央企省企名企等优强企业的对接合作，主动承接产业转移，着力引进区域总部企业、行业领军企业。科学规划，做实做强“蓝宁道新江”加工贸易走廊。扩大对外经贸合作，鼓励水电、采矿、农产品加工、装备制造等产业抱团“走出去”，大力推动外贸进出口基地建设，继续推进外贸企业“破零倍增”工作，重点扶持“五十百”外贸主体做大做强。全面构筑大通关。加快湖南农副产品集中验放场项目封关运行，争取叠加“保税物流”功能。做好永州公路口岸项目二期规划设计，加快交易区建设，争取成为国家进境水果指定口岸和一类公路口岸。长沙海关永州办事处挂牌运作，永州内陆无水港项目进入实质性操作。支持有条件、有需求的县区和进出口企业在本地本企业建设监管仓和保税仓。开通港澳直通车，主动对接上海、广东自贸区和中国—东盟自贸区，把沿海、沿边口岸的功能延伸和拓展到永州。加快电子口岸建设，实行“一次申报、一次查验、一次放行”的通关模式。

突出创新驱动。加强以企业为主体的创新平台建设，新建省级企业工程技术研究中心1家，市级企业工程技术研究中心3家，实施省以上科技计划50项以上，新认定高新技术企业5家以上。支持永州国家农业科技园及宇秀科技、异蛇科技、和广生物等企业与科研院校紧密合作，力争在食用菌、异蛇、油茶、柑橘等领域至少创建一个国家级研发中心、检测中心或实验室。加快湘南进出口食品（农副产品）检测中心项目建设，努力建成国家级重点实验室。加快创新成果转化，加强汽车及零部件、动力电池等技术攻关，通过产业化的创新来培育新的经济增长点。牢固树立人力资源至上的思维，加强创新创业人才的引进、培养和使用，营造鼓励创新创业的人才成长环境。

　　（三）扎实推进新型城镇化，统筹区域城乡发展

　　做大做强中心城市。深入实施中心城区三年扩容提质计划，今年启动重点项目220个，完成投资84亿元。抓好大创建，巩固提升创建国家卫生城市成果，构建常态、长效机制，继续抓好国家历史文化名城申报工作，加大国家交通管理模范城市创建力度，努力实现创建国家森林城市目标。推进大扩容，加快滨江新城基础设施建设，实施市民中心、文化艺术中心、艺术综合体等项目，启动东部新城路网建设。加快祁阳、东安、双牌融城步伐，推进北部潇湘城市群建设。实施大提质，充分利用“两房两棚”、“两供两治”政策，加快老城区改造提质，抓好冷水滩湘江西岸、零陵区潇水西路等棚户区和城中村集中连片改造、闲置土地清理、控违拆违等工作。实施永州大道配套工程，加快春江路、九嶷大道、梧桐路、凤凰路、南津路、潇水路等提质改造，推进断头路、安置小区建设，新建11个小游园。抓好曲河水厂、市水质净化中心、下河线污水处理厂扩容提质，推进垃圾处理工程建设。加快建设冷水滩至零陵快速公交(BRT)。坚持建管并重，提高管理水平，提升城市品质。

　　做美做活县城。把县城作为推进新型城镇化和加快县域经济发展的重要平台，完善规划体系，推进“多规合一”，切实加强市政公用设施和公共服务设施建设，全面提升县城综合承载能力和公共服务水平。放开户籍管理，推进农业转移人口市民化。深入开展县城提质达标行动，积极推进文明卫生创建活动。加强特色县域经济重点县建设。支持祁阳、道县开展撤县建市基础性工作。

　　做精做特小城镇。大力推进品质活力示范乡镇建设，在着力抓好15个全国重点镇和省级示范乡镇的同时，每个县区集中力量建设3至5个品质活力示范乡镇。积极培育一批工业强镇、商贸重镇和旅游名镇，加快发展省际边界口子镇。加强村庄规划，规范农民建房，整治农村环境，建设美丽乡村，办好13个省级新农村建设示范点，抓好520个村的人居环境整治，打造20个省级美丽乡村。

　　（四）进一步完善基础条件，为稳增长提供坚实支撑

　　完善交通网络。积极推动湘桂线电气化改造、洛湛铁路扩能改造工程早日开工，抓紧湘桂铁路新建高溪市至永州站三线前期工作，争取湘桂高铁动车继续加密，力争兴永郴赣铁路纳入国家建设规划。加强与广西沟通对接，争取道贺高速、厦蓉高速广西段早日联通，做好衡永高速公路前期工作。完成湘江永州千吨级航道改造和祁阳、冷水滩、零陵3个千吨级码头项目前期。推进零陵机场迁建前期工作，稳步发展民航业。进一步完善市内公路网络，将207国道双牌至零陵段作为一级公路进行改造，推进东安至冷水滩快速通道等一批项目开工建设，207国道双牌至道县段等一批在建项目竣工通车。完成农村通畅工程600公里，新增137个行政村，实现100%行政村通畅，建成农村招呼站230个。

　　完善水利网络。扎实抓好涔天河水库扩建工程，积极稳妥做好库区移民应急度汛和搬迁安置工作，推动涔天河灌区、毛俊水库开工建设，加快芦江水库、金钩挂水库建设进度。推进4座中型水库除险加固工程，完成10个中小河流治理及“四水”治理、6个全国小农水重点县、17座小一型和92座小二型水库除险加固、5个水土流失综合治理、1个中型灌区续建等项目建设。新解决81.5万农村人口饮水安全问题。

　　完善能源网络。加快建设神华国华永州火电一期工程，积极做好二期立项前期工作。抓好新田220千伏变电站和冷水滩岚角山、东安芦洪市、宁远城南、祁阳西区4个110千伏变电站建设。加强中心城区电网建设改造，提高城市供电可靠性。完成258个行政村农网改造和30114户“低电压”整治。加强新型清洁能源点建设，把发展风电作为扩大投资的重点，力争江华东田、江永黄甲岭、零陵石岩头3个总装机15万千瓦风电项目建成投产，江华界牌、宁远盘洞口、蓝山四海坪、道县洪塘营、江永上江圩5个总装机28万千瓦风电项目开工建设。积极推进分布式光伏电站建设。抓住省里实施“气化湖南”机遇，争取支持，加快推进天然气输气管网建设。

　　完善信息网络。大力实施智慧城市创建计划，借助大数据、云计算平台，推进电子政务、公共服务、社会治理三位一体数字化网络平台建设，抓好4G 网络、宽带提速、基站建设等项目。深入实施两化深度融合“111”工程和省政府促进信息消费十大工程，完成2家数字示范园区、3家省级示范企业、24个市级两化融合重点项目以及200家数字企业创建任务。

　　（五）保护好湘江源头，大力加强生态文明建设

　　切实加强生态保护。严格实施主体功能区规划，扎实推进绿色永州建设，大力开展植树造林和封山育林，坚决打好绿化裸露山体歼灭战。建立健全生态补偿机制，推进湘江源头区域国家生态文明先行示范区建设，抓好江华、蓝山、新田、宁远、双牌国家主体功能区建设试点示范，强化对江华涔天河、双牌日月湖、东安紫水三个国家湿地公园的管理，力争新增1至2个国家湿地公园。

　　深入开展污染防治。严格环保执法，强化水污染治理，全面完成湘江保护与治理第一个“三年行动计划”，重点加强工业废水、生活污水、养殖污染等源头防控。强化大气污染监测和防治，积极推进湘江纸业异地搬迁改造，努力保持空气质量优良。推进农村生活垃圾处理、改水、改厕和改气工作。强化矿山开采秩序整治，组织实施好石期河流域等矿区综合治理工程。全面完成重金属污染防治“十二五”规划。

　　切实抓好节能减排。大力发展生态型循环经济，突出分类施策和减量调整，严格执行节能节地节水和环境、技术、安全等市场准入标准，坚决淘汰高能耗、高排放、高污染、低效益的落后产能。加快推进污水、垃圾处理设施和配套管网建设，加强对工业园区和水泥、造纸、涉重涉危行业的监督检查。

　　（六）保障和改善民生，促进社会事业协调发展

　　扎实办好民生实事。切实抓好省里提出的 “两房两棚”、“两供两治”、城镇就业、农村公路建设、农贸市场改造等15个方面、24件为民办实事项目，分解任务，明确责任，全面落实。继续下大力办好棚户区改造、加强城市小区管理、农村环境污染治理、抓好放心食用油和米粉、“五小水利”工程建设、确保社会安全等市定六件惠民实事，确保全面完成和超额完成年度目标任务。

　　大力推进创新创业。坚持就业优先，推动大众创业、万众创新，鼓励支持高校毕业生、城镇失业人员、被征地农民、退役军人、外出务工人员等群体自主创业、返乡创业，力争全年新增城镇就业5.5万人、农村劳动力转移就业6万人，高校毕业生就业率稳定在85%以上。加强政府公共就业服务，加大对就业困难人员的帮扶力度。推进创业型城市建设，建立一批大学生创业园和创业孵化基地。健全面向全体劳动者的职业培训制度，构建劳动者终身职业培训体系。

　　完善社会保障体系。提升社会保障水平，完成五险参保576.6万人次，社会保障卡发放到所有参保对象。提高城乡低保、农村五保救助标准，加强城乡低保资金监管，保障特殊困难群众基本生活。精心组织实施机关事业单位工作人员养老保险制度改革。规范保障性安居工程建设、分配和管理。推进社会养老服务，引导民间资本投入健康养老事业，发展成为新的增长点。

　　促进教育优先发展。新增城区义务教育学位1.9万个，其中中心城区6000个，建设义务教育合格学校48所，新建农村公办幼儿园22所。编制和实施全市第二轮学前教育三年行动计划。加强名校名师名校长和师德师风师资建设，不断提高教育教学质量。鼓励支持社会力量办学。推进家庭教育示范县创建。加强教育信息化工程建设。

　　推进文化发展繁荣。切实加强社会主义核心价值观教育，广泛开展群众性精神文明创建活动。继续实施文化惠民工程，完善市、县、乡、村四级文化设施网络，加强濂溪故里、涧岩头、上甘棠等文化遗产项目规划建设，抓好祁剧、江永女书等非物质文化遗产和传统村落的保护，推进广播“村村响”、电视“户户通”。精心做好省政府第四次公祭舜帝大典相关工作。

　　统筹发展医药卫生等社会事业。深化医药卫生体制改革，全面推进公立医院综合改革，逐步建立分级诊疗制度，抓好重大疾病防控，支持社会办医，鼓励发展名医名科名院，促进基本公共卫生服务均等化。加强人口和计划生育工作，狠抓出生人口性别比例综合治理，全面落实计划生育利益导向政策。抓好第二次全国地名普查工作。发展竞技体育，开展全民健身。发展老龄事业，保障妇女、儿童权益，关心青少年成长，关爱留守儿童、留守妇女和留守老人。支持发展慈善事业，做好残疾人基本公共服务和残疾预防工作。做好“双联”工作。实施精准扶贫，加大产业扶贫力度，加快基础设施建设，加强基本公共服务，进一步改善贫困地区群众生产生活条件。加大民族政策落实力度，加快少数民族地区、边远地区、革命老区、林区、库区经济发展。

　　维护社会和谐稳定。健全完善立体化防控体系，依法严厉打击“两抢一盗”、“三强”、黄赌毒、非法集资等各类违法犯罪活动，提高防恐防爆能力。推进警务体制改革，推动警力下沉、主动警务和智慧公安建设，加强农村社会治安综合整治。完善人民调解、行政调解、司法调解联动工作体系，做好社区矫正和法律援助工作，深入开展“六五”普法。加快建设市、县网上信访信息系统，继续推进领导干部下访包案、积案化解等工作。健全安全生产工作机制和责任体系，深入开展“四大三基”三年行动计划，加快推进隐患排查治理体系和安全生产标准化建设，完善突发事件应急管理和防灾减灾体系，提升对突发事件综合处理能力。大力整顿和规范市场秩序，严厉打击制售假冒伪劣行为。切实强化食品药品监管，深入开展“打黑除非”整治行动。创新虚拟社会管理，依法规范网络行为。发挥礼序家规、乡规民约在社会治理中的积极作用。

　　科学编制“十三五”规划是今年的一项重大任务。要本着对全市人民和永州长远发展极端负责的态度，深入研究新常态下的经济发展规律，准确把握未来发展面临的机遇和挑战，科学研究提出今后五年的发展目标和主要任务，特别是要策划包装一批重大项目，争取列入国家和省里“十三五”规划笼子。

　　扎实推进国防和后备力量建设。加强国防动员应急应战一体化建设，推进后备力量建设转型发展，深入开展双拥共建和“双带双促”活动，加强军事和人防设施保护工作，切实维护保障部队和军人军属合法权益，充分发挥部队、民兵预备役人员在参建参治中的排头兵和生力军作用。

　　四、推进依法治市，努力开创政府自身建设新局面

　　经济发展进入新常态，政府工作面临的任务更加繁重、责任更加重大。各级政府及其工作人员要适应新常态，把握新要求，以更大的决心和勇气、更实的举措和办法，提振精气神，汇聚正能量，切实加强政府自身建设。

　　强化法治意识，提高依法行政能力。认真落实市人大及其常委会的决议决定，自觉接受市人大及其常委会的监督，支持人民政协参政议政，及时办理人大代表议案、建议和政协委员提案。坚持依法科学民主决策，实施信访评估、社会稳定风险评估、行政决策风险评估“三评合一”，建立重大决策终身责任追究制度及责任倒查机制。完善政府常务会议学法、政府及部门法律顾问、规范性文件审查备案等制度。推进重大决策部署、重要民生事项、重大投资项目和公共资源交易等领域的信息公开，提高工作透明度，加强诚信政府建设。推进综合执法改革，规范行政执法行为。

　　强化宗旨意识，提高为民服务能力。巩固扩大党的群众路线教育实践活动成果，进一步完善领导干部带头深入基层调研、直接联系群众和服务群众制度，着力构建反“四风”常态化长效化机制。切实加强政务服务、社区服务、电子政务、12345热线等平台建设，规范权力运行，推行阳光行权，提高办事效率。持续开展电视问政、网络问政，积极回应群众诉求。关心爱护基层干部，进一步改善乡镇、街道、社区的办公和生活条件。

　　强化责任意识，提高落实执行能力。认真落实市委关于加强干部队伍执行力建设的意见，全面推行领导干部实绩卡和执行力卡管理。积极推行“经济工作项目化、项目工作责任化”，层层量化指标、细化任务、强化责任，确保重大项目、重点工作不折不扣落到实处。改进文风会风，精简会议文件，减少迎来送往，把更多的时间和精力用在抓落实上。强化目标刚性约束，严格政府绩效考核，形成鲜明导向体系，真正以目标引领行动，以执行推动落实。

　　强化创新意识，提高攻坚克难能力。牢固树立底线思维，凡事从守住底线开始，做到有备无患，牢牢把握主动权。切实强化问题导向，对影响工作进展的关键问题、制约长远发展的重大问题、群众反映强烈的热点难点问题，研究解决方案，制定实施计划，实行挂图作战、跟踪问效。深入推进改革创新，善于运用市场手段、改革办法和创新思维，发现捕捉新机遇，科学破解新挑战，加快培育新动力，努力谋求新发展。

　　强化廉政意识，提高廉洁自律能力。建立健全严格的财政预算、核准和审计制度，管好公共资金、国有资产和国有资源。加强政府内部权力制约，对权力集中的部门和岗位实行分事行权、分岗设权、分级授权、定期轮岗。完善政府内部层级监督和专门监督，强化审计监督。严格落实廉洁从政规定和政府工作规则，科学有效预防腐败，坚决果断惩治腐败，切实做到干部清正、政府清廉、政治清明。

　　各位代表，新常态蕴含新机遇，新常态催生新发展。让我们紧密团结在以习近平同志为总书记的党中央周围，在中共永州市委的坚强领导下，惟公惟民不惜力，尽职尽责不松劲，克勤克俭不懈怠，锐意进取，奋发有为，为加快富民强市步伐、建设品质活力永州而努力奋斗！
